# Histology-Specific Survival in Penile Squamous Cell Carcinoma: A SEER-Based Study Highlighting Human Papillomavirus Status and Prognostic Subtypes

**DOI:** 10.3390/cancers17223715

**Published:** 2025-11-20

**Authors:** Yudai Ishiyama, Arjun Venkatesh, Reynier David Rodriguez Rosales, Jean-Pierre Kanumuambidi, Mark Bandyk, K. C. Balaji

**Affiliations:** 1 Department of Urology, University of Florida Jacksonville, 655 8th St W, Jacksonville, FL 32209, USA; yuishi0831@gmail.com (Y.I.);; 2 Department of Urology, Tokyo Women’s Medical University, 8-1 Kawada-cho, Shinjuku-ku, Tokyo 162-0054, Japan; 3 School of Medicine, St. George’s University, 1 True Blue Drive, St. George’s P.O. Box 7, Grenada

**Keywords:** human papillomavirus viruses, penile neoplasms, SEER program, squamous cell cancer

## Abstract

**Background:** Penile squamous cell carcinoma (SCC) is now categorized based on human papillomavirus (HPV) status as HPV associated (SCCa) or HPV-independent. While most HPV-independent cases are of the usual type (Usual-SCCi), the prognostic significance of this subclassification—particularly between Usual-SCCi and other SCC HPV-independent variants (Variant-SCCi)—remains unclear. **Materials and Methods:** Using SEER data (2000–2021), patients with penile SCC were categorized into Usual-SCCi, Variant-SCCi (verrucous, papillary, and sarcomatoid), and SCCa groups. Cancer-specific survival (CSS) was evaluated using Kaplan–Meier estimates and Cox proportional hazard regression adjusted for demographic and clinical factors, with Firth’s penalized likelihood correction applied when appropriate. Temporal trends and subtype-specific outcomes were also analyzed. **Results:** Among 6332 patients, 5706 (90.1%) were Usual-SCCi, 416 (6.6%) were Variant-SCCi, and 210 (3.3%) were SCCa. Overall, 5-year CSS (proportion of patients (%) [95% CI]) was the highest in Variant-SCCi (90.9% [87.8–94.0]), compared to Usual-SCCi (73.7% [72.4–75.0]) and SCCa (71.5% [63.3–80.8). Within Variant-SCCi, verrucous SCC had the most favorable outcome (94.1% [91.3–97.0]), followed by papillary (81.9% [68.2–98.4]) and sarcomatoid (63.4% [47.4–84.7]). Multivariable analysis confirmed verrucous SCC as being independently associated with significantly lower cancer-specific mortality (HR: 0.31 [95% CI, 0.20–0.45]; *p* < 0.001). Time-stratified analysis revealed that in the latter half of the study period, SCCa outcomes improved markedly, and no statistically significant differences in CSS were observed among the SCCa, papillary, and Usual-SCCi groups, leaving verrucous SCC as the only subtype with consistently superior survival (*p* < 0.001). **Conclusions:** The CSS of SCCa improved over time to become equivalent to those of the SCCi subtypes except for the verrucous SCCi variant, which had a significantly better CSS. Our study clearly demonstrates a temporal shift toward improved CSS in the SCCa subtype, which may be related to increased awareness following recognition as a separate entity in the WHO 2022 classification and other factors.

## Simple Summary

This population-based study of 6332 penile SCC cases revealed that variant HPV-independent subtypes, particularly verrucous carcinoma, had superior cancer-specific survival. Our study clearly demonstrates a temporal shift toward improved survival for HPV-associated SCC to match the usual type and papillary subtypes, but verrucous SCC consistently showed the most favorable prognosis.

## 1. Introduction

Squamous cell carcinoma (SCC) is the most prevalent pathology among penile malignancies, followed by variants and other malignancies, such as basal cell carcinoma, sarcomas, melanoma, extramammary Paget disease, and metastases from distant primaries [1,2,3]. SCC accounts for approximately 95% of all penile malignancies and is further categorized based on human papillomavirus (HPV) status, as defined by the 2022 World Health Organization (WHO) classification system [4]. The recognition is that molecular alterations that drive carcinogenesis are distinct between the two categories. HPV-associated SCC (SCCa), including basaloid, warty, clear cell, and lymphoepithelioma-like types, arises from the infection of basal epithelial cells by HPV, typically through microtrauma. The viral DNA integrates into the host genome, leading to the overexpressions of oncoproteins E6 and E7. E6 promotes the degradation of p53, thereby inhibiting apoptosis, while E7 inactivates the Rb protein, driving cell cycle progression and uncontrolled proliferation [5]. The carcinogenic mechanisms of HPV-independent SCCs, including squamous cell carcinoma (the usual type (Usual-SCCi)), verrucous carcinoma (papillary and sarcomatoid types)—herein referred to as HPV independent, and other variant subtypes (Variant-SCCi), are less understood but are often associated with chronic inflammation [6]. This inflammatory process generates reactive oxygen species and nitrogen intermediates and activates COX-2, leading to DNA damage, proliferation, angiogenesis, and invasion [7,8]. While the aforementioned classification distinguishes SCCa and HPV-independent SCCs, Usual-SCCi, often regarded as “pure” SCC, is classified under HPV-independent and comprises up to 80% of such cases. The prognostic relevance of the recent HPV-based classification remains unclear and, in particular, whether differences exist between Usual-SCCi and Variant-SCCi has never been investigated [9]. Furthermore, trends in the prognosis of these groups of patients over extended periods of time are rarely reported.

The rarity of penile cancer makes investigating epidemiology and outcomes challenging. The Surveillance, Epidemiology, and End Results (SEER) database has been instrumental in overcoming this limitation, enabling studies that elucidate demographic patterns, treatment approaches, and emerging trends in penile cancer management [10,11,12,13,14]. Recent SEER-based studies have reported descriptive characteristics and survival outcomes of non-SCC penile cancers compared to those of SCC [15]. Prognostic nomograms incorporating HPV-related factors have also been reported [16]. However, no study to date has specifically investigated survival differences among Usual-SCCi, Variant-SCCi, and SCCa using this database.

In this context, the main aim of the current study is to compare the survival rates of the three novel pathological categories, namely, Usual-SCCi, Variant-SCCi, and SCCa. We also aim to describe the outcomes of specific subtypes and assess the changes over time.

## 2. Materials and Methods

### 2.1. Data Source

Study data were obtained from Surveillance, Epidemiology, and End Results (SEER) research plus data, 17 registries (2000–2021), using SEER*Stat software (Version 8.4.4). The Surveillance, Epidemiology, and End Results (SEER) database is a comprehensive source of cancer incidence and survival data in the United States. Covering approximately 48% of the population, SEER provides detailed cancer statistics essential for studying cancer epidemiology at the population level. Survival data were obtained from the most recently available full-field SEER registry (SEER 17), with patients from the years 2000 to 2021. Patients with a primary site coded as the penis were identified based on the International Classification of Diseases for Oncology, Second Edition (ICD-O-2), site codes from C60.0 to C60.9. Histology was classified using the International Classification of Diseases in Oncology, Third Edition (ICD-O-3) and further categorized into four groups based on the 2022 WHO Penile Cancer Classification as follows:

1. HPV independent, usual type SCC (Usual-SCCi);
2. HPV independent, other variant subtypes (Variant-SCCi): verrucous carcinoma, papillary and sarcomatoid types;
3. HPV-associated SCC (SCCa): basaloid, warty, and clear cell. No cases were listed for lymphoepithelioma-like types.

**Figure 1:**
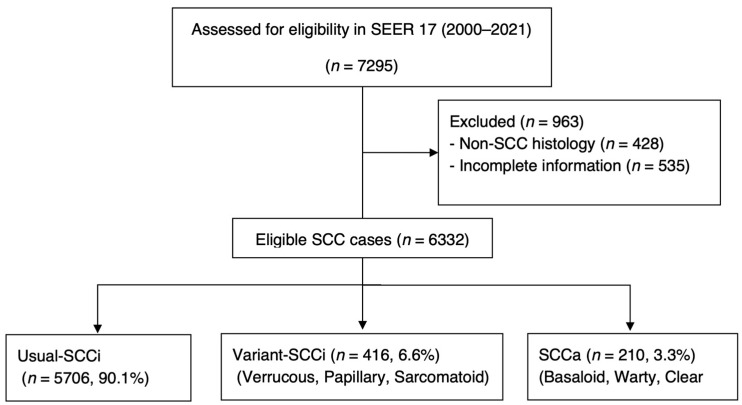
A CONSORT-style flow diagram illustrating the case selection process. SCCa, human-papillomavirus-associated squamous cell carcinoma; Variant-SCCi, HPV-independent squamous cell carcinoma variant subtypes; SEER, Surveillance, Epidemiology, and End Results; SCC, squamous cell carcinoma; Usual-SCCi, usual-type squamous cell carcinoma.

The “HPV-associated” status was inferred from histologic coding rather than confirmed by molecular or immunohistochemical testing, as the SEER database does not provide HPV or p16 IHC confirmation. Additional patient information collected included the year of diagnosis, age at diagnosis, race, extent of disease (EOD), type of surgery, radiation status, chemotherapy status, and survival data. EOD was obtained from the combined summary stage (2004+), SEER combined summary stage 2000 (2004–2017), SEER historic stage A (1973–2015), and summary stage 2018 classifications. EOD was manually reclassified into four categories: localized, regional, distant, and unknown. Surgical interventions were reclassified into five categories: none/unknown, local, partial, total, and extensive based on ‘RX Summ--Surg Prim Site’ information. Survival data included survival months and cause of death, classified as cancer-specific mortality (CSM; death attributable to penile SCC) or other-cause mortality (OCM; death attributable to other causes) based on the SEER mortality code. From the original data on 7295 patients extracted according to the inclusion criteria above, those with non-SCC histology (*n* = 428) or incomplete clinical or pathological information (*n* = 535) were excluded from the final analysis (Figure 1). Patients with available data for all the variables (including those recorded as ‘unknown’) were included. The institutional review board determined that this study qualified as non-human-subject research (NH00045999) and granted approval for its conduct. The study was conducted in accordance with the Declaration of Helsinki.

### 2.2. Study Design and Statistical Analyses

The primary outcome of interest was cancer-specific survival (CSS) after accounting for other-cause mortality. CSS was estimated using the Kaplan–Meier method, and differences were assessed using the log-rank test. The study cohort was divided into three groups based on histology classification (Usual-SCCi, Variant-SCCi, and SCCa) and compared across groups using the log-rank test. Further analyses were conducted to assess the proportion and the CSS of specific subtypes within Variant-SCCi or SCCa, depending on the number of samples included, as well as the differences of the outcomes over the first (2000–2010) and second (2011–2021) halves of the study period. Univariate and multivariate Cox proportional hazard regression models were employed to assess the associations between the histological classification and other clinical variables with CSS. In analyses involving subgroups with limited sample sizes (*n* ≤ 50), Firth’s penalized likelihood correction was utilized to mitigate potential small-sample bias and improve the model’s stability. Variables included the year of diagnosis (2000–2005, 2006–2010, 2011–2015, and 2016–2021), age at diagnosis, race (White, Black, Asian or Pacific Islander, American Indian/Alaska Native, and unknown), EOD (localized, regional, distant, and unknown), radiation status, and chemotherapy status. The results of the Cox proportional hazard regression models were reported using hazard ratios (HRs) with 95% confidence intervals (CIs). Continuous variables were analyzed using the Mann–Whitney U-test, while categorical variables were analyzed using chi-squared or Fisher’s exact tests, as appropriate, with 95% confidence intervals (CIs). All the statistical analyses were performed using R software (The R Foundation for Statistical Computing, Vienna, Austria), with statistical significance set at *p* < 0.05.

## 3. Results

### 3.1. Patient Characteristics

A total of 6332 patients were included in the formal analysis. Among these, 5706 (90.1%) were classified as Usual-SCCi, 416 (6.6%) as Variant-SCCi, and 210 (3.3%) as SCCa. Significant differences were noted among the three histology categories across all the collected variables except for race (*p* = 0.621; other, *p* < 0.001). In terms of the year of diagnosis, SCCa was most frequently diagnosed at the last term (between 2016 and 2021), accounting for 64.3% of the cases in this histological category. Patients in the Variant-SCCi group had the lowest median age at diagnosis (65.0 [IQR 54.0, 75.3]) compared with those in the Usual-SCCi (68.0 [57.00, 77.00]) and SCCa (69.5 [60.00, 78.00]) groups. The Variant-SCCi group was also the most often diagnosed as having localized disease (*n* = 314, 75.5%), the highest utilization of local surgery (*n* = 154, 37.0%), and the lowest usage of radiation therapy (*n* = 22, 5.3%) or chemotherapy administration (*n* = 23, 5.5%). Other baseline characteristics, including race, extent of disease, and treatment details, are summarized in Table 1.

### 3.2. CSS According to Three Histology Categories

**Figure 2:**
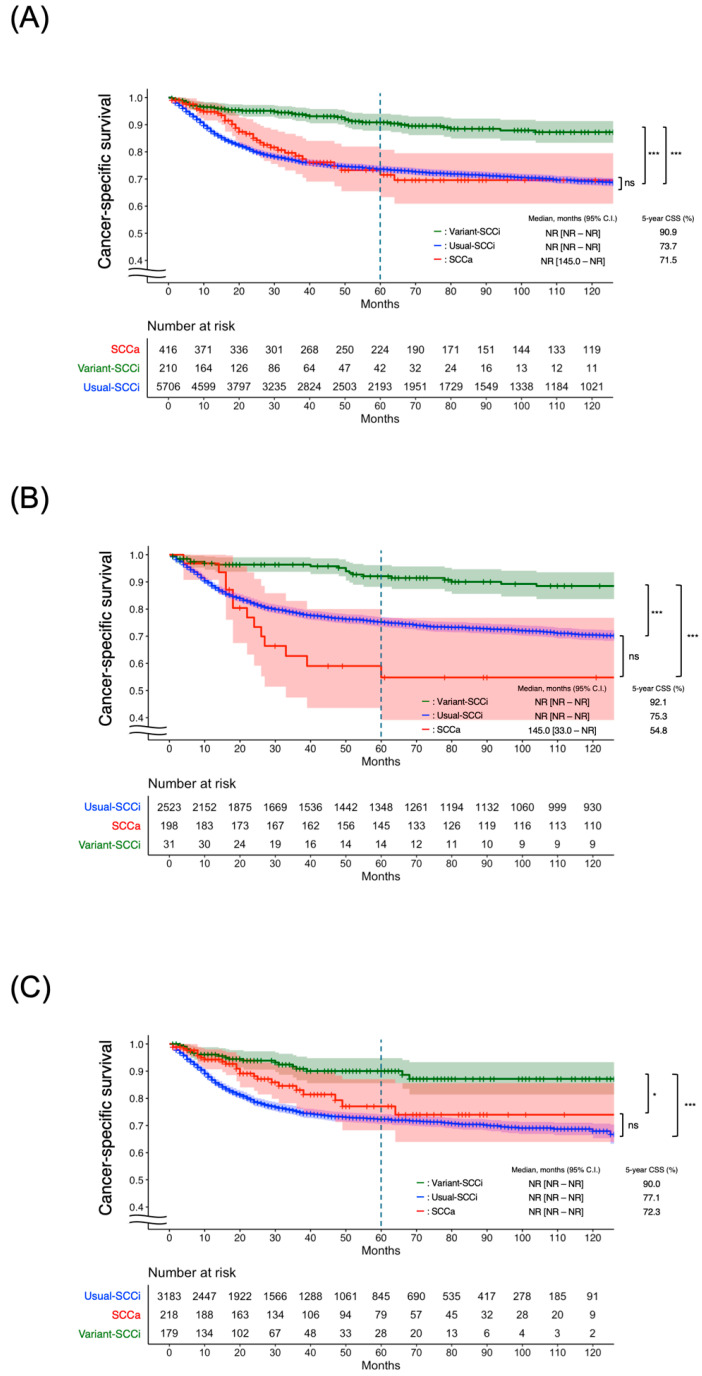
Cancer-specific survivals by the three histological groups: (**A**) entire study period; (**B**) 2000–2010; (**C**) 2011–2021. Statistical significance is indicated as follows: ns, not significant (*p* > 0.05); * *p* < 0.05; *** *p* < 0.001. CI, confidence interval; CSS, cancer-specific survival; SCCa, human-papillomavirus-associated squamous cell carcinoma; Variant-SCCi, HPV-independent squamous cell carcinoma variant subtypes; NR, not reached; Usual-SCCi, usual-type squamous cell carcinoma.

During the median 39.0 [IQR 13.0, 95.0] months of follow-up, 1478 (23.3%) cases of cancer-specific death occurred (Figure S1). At a landmark of 5 years, 1318 cases of cancer-specific death were observed, and the CSS rate was 75.0%, [95% CI 74.0–76.0]. The median survival was not reached in any of the three cohorts (Usual-SCCi, Variant-SCCi, and SCCa). The 5-year CSS (proportion of patients (%) [95% CI]) was significantly longer in the Variant-SCCi group (90.9% [87.8–94.0]) compared with those in all the Usual-SCCi (73.7% [72.4–75.0]) and SCCa (71.5% [63.3–80.8]) groups (Figure 2A). In the subgroup analyses according to the year of diagnosis, the 5-year CSS was higher in the Variant-SCCi (92.1% [88.2–96.2]) group, followed by the Usual-SCCi (75.3% [73.5–77.1]) and SCCa (54.8% [39.1–76.8]) groups in the first half of the study period (Figure 2B). There was no statistically significant difference in CSS between the Usual-SCCi and SCCa groups. In the second half of the study period, the 5-year CSS was the highest in the Variant-SCCi group (90.0% [85.5–94.8]), followed by the SCCa (77.1% [68.2–87.0]) and Usual-SCCi (72.3% [70.5–74.2]) groups. Again, no statistically significant difference was observed in CSS between the SCCa and Usual-SCCi groups (Figure 2C). Additional stratification of the tumor stage and treatment distributions by era is shown in Supplemental Table S1.

### 3.3. CSS Sub-Stratified by Components of Variant-SCCi and SCCa

**Figure 3:**
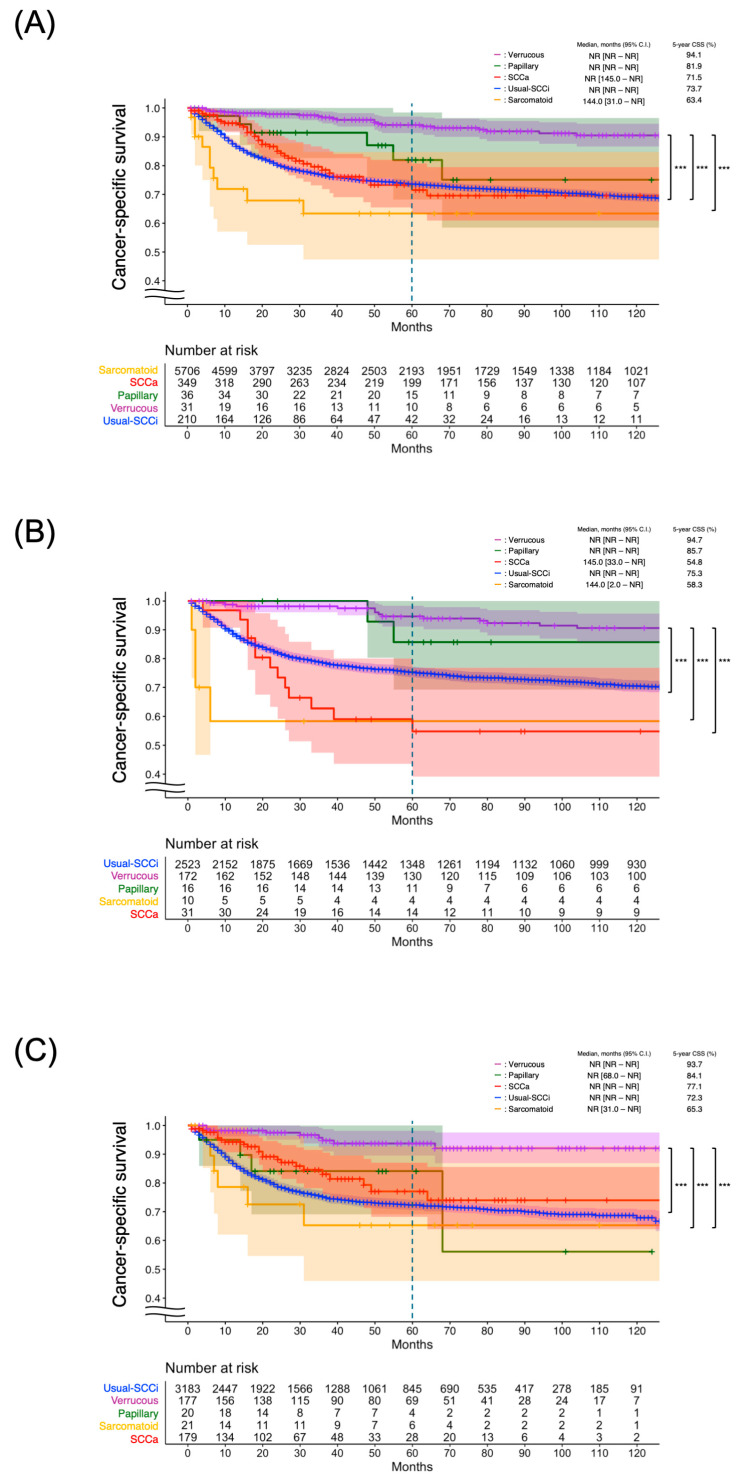
Cancer-specific survivals by five histological groups (Usual-SCCi, SCCa, verrucous carcinoma, papillary carcinoma, and sarcomatoid carcinoma). The late decline observed in the papillary group likely reflects instability due to the limited number of patients at risk in the later follow-up period: (**A**) entire study period; (**B**) 2000–2010; (**C**) 2011–2021. Statistical significance is indicated as *** *p* < 0.001. CI, confidence interval; CSS, cancer-specific survival; SCCa, human-papillomavirus-associated squamous cell carcinoma; Variant-SCCi, HPV-independent squamous cell carcinoma variant subtypes; NR, not reached; Usual-SCCi, usual-type squamous cell carcinoma.

According to the finding that the Variant-SCCi group exhibited significantly longer survival than the other two groups, we conducted a further analysis by subclassifying this group into its three original histological subtypes—verrucous carcinoma, papillary carcinoma, and sarcomatoid carcinoma—along with Usual-SCCi and SCCa for comparison. Verrucous carcinoma showed the longest median CSS (median months, not reached (NR) [95% CI NR-NR]) and highest 5-year CSS (94.1% [91.3–97.0]) among the five histological subtypes, with significantly longer median times and higher 5-year rates than those observed in the sarcomatoid (median months, 144.0 [95% CI 31.0-NR], 5-year CSS: 63.4% [47.4–84.7]), SCCa (median months, NR [95% CI 145.0-NR], 5-year CSS: 71.5% [63.3–80.8]), and Usual-SCCi (median months, NR [95% CI NR-NR], 5-year CSS: 73.7% [72.4–75.0]) groups. The median time and 5-year CSS were NR [95% CI NR-NR] and 81.9% [68.2–98.4] in the papillary group, with no significant differences compared to any other group (Figure 3A).

When stratified by study period, the pattern of CSS differed between the first and second halves. In the first half, the 5-year CSS was the highest in the verrucous group (94.7% [91.1–98.3]), followed by papillary (85.7% [69.2–100.0]), Usual-SCCi (75.3% [73.5–77.1]), sarcomatoid (58.3% [34.0–100.0]), and SCCa (54.8% [39.1–76.8]) groups. Statistically significant differences were observed between verrucous and each of sarcomatoid, SCCa, and Usual-SCCi (all *p* < 0.05) (Figure 3B). In the second half, verrucous again showed the highest 5-year CSS (93.7% [89.5–98.1]), followed by papillary (84.1% [69.1–100.0]), SCCa (77.1% [68.2–87.0]), Usual-SCCi (72.3% [70.5–74.2]), and sarcomatoid (65.3% [45.9–92.8]). Although the papillary group showed a relatively high 5-year CSS, its survival curve declined after 70 months and ultimately became the lowest among all the subtypes at 120 months. This likely reflects statistical instability caused by the small number of patients at risk during the late follow-up period. Significant differences remained between the verrucous group and each of sarcomatoid, SCCa, and Usual-SCCi in the second half (Figure 3C).

For SCCa, it was observed that the majority (92.9%) were basaloid carcinoma, with 6.7% being warty carcinoma and only one case of clear cell SCC. As such, no sub-stratification analyses were performed.

A sensitivity analysis was conducted assuming that 10% of the Usual-SCCi cases were HPV positive. A 10% assumption was made based on a previous study reporting that 16.3% of the Usual-SCCi cases showed p16 positivity [17]. Under this assumption, the direction and statistical significance of the associations remained unchanged (Supplemental Figure S2).

### 3.4. Univariable and Multivariable Analyses

Given the observed survival differences among the HPV-independent subtypes, we conducted a Cox proportional hazard analysis using histology, defined in a detailed manner as five separate subtypes: Usual-SCCi, SCCa, verrucous carcinoma, papillary, and sarcomatoid. Univariable analysis (HR [95%CI]) revealed that histology (verrucous, 0.22 [0.14–0.32]; SCCa, 0.88 [0.63–1.21]; papillary, 0.65 [0.27–1.30]; sarcomatoid, 1.81 [0.96–3.09] in reference to Usual-SCCi, *p* < 0.001, 0.460, 0.245, and 0.067, respectively), EOD (regional, 3.16 [2.82–3.55]; distant, 14.2 [11.90–16.95]; unknown, 2.07 [1.59–2.70] in reference to local, all *p* < 0.001), type of surgery (partial, 1.79 [1.55–2.07]; total, 3.33 [2.79–3.98]; extensive, 3.54 ([2.77–4.51]; none/unknown, 4.12 [3.44–4.92] in reference to local, all *p* < 0.001), radiation status (yes, 2.49 [2.17–2.86] in reference to no/unknown, *p* < 0.001), and chemotherapy (yes, 3.14 [2.78–3.54] in reference to no/unknown, *p* < 0.001) were significantly associated with CSS. Among demographic variables, age (1.01 [1.01–1.02] per year increase; *p* < 0.001), diagnosis year (2016–2021, 1.21 [1.04–1.41] in reference to 2001–2005, *p* = 0.011), and race (Black vs. White, 1.20 [1.02–1.42], *p* = 0.028; unknown vs. White, 0.14 [0.03–0.56], *p* = 0.005) were also significantly associated with CSS, whereas Asian (0.90 [0.69–1.16]; *p* = 0.420) and Native American/Alaskan Native (1.13 [0.69–1.85]; *p* = 0.623) were not (Table 2).

Multivariable analysis (HR [95% CI]) revealed that histology (verrucous, 0.31 [0.20–0.45]; SCCa, 0.78 [0.55–1.06]; papillary, 0.82 [0.34–1.62]; sarcomatoid, 1.64 [0.86–2.80] in reference to Usual-SCCi, *p* < 0.001, 0.112, 0.594, and 0.123, respectively), extent of disease (EOD) (regional, 2.51 [2.21–2.85]; distant, 8.11 [6.61–9.91]; unknown, 1.53 [1.15–2.00] in reference to local, all *p* < 0.01), and type of surgery (partial, 1.16 [1.00–1.36]; total, 1.77 [1.46–2.13]; extensive, 1.85 [1.43–2.38]; none/unknown, 2.25 [1.86–2.74] in reference to local, *p* = 0.055, < 0.001, < 0.001, and < 0.001, respectively) were significantly associated with CSS, except for partial resection (*p* = 0.055). Among the treatment factors, chemotherapy (1.50 [1.29–1.73]; *p* < 0.001) and radiation (1.26 [1.08–1.46]; *p* = 0.004) were associated with higher cancer-specific mortality. Regarding demographic variables, age (1.02 [1.01–1.02] per year; *p* < 0.001) and race (unknown vs. White, 0.18 [0.04–0.52]; *p* < 0.001) were significant, whereas Black (1.17 [0.98–1.38]; *p* = 0.076), Asian (0.86 [0.66–1.10]; *p* = 0.230), and Native American/Alaskan Native (1.18 [0.70–1.85]; *p* = 0.518) were not. The diagnosis year showed no significant association across all the periods (2011–2015, 1.06 [0.91–1.23]; 2016–2021, 1.14 [0.97–1.34]; vs. 2001–2005; all *p* > 0.05) (Table 3).

## 4. Discussion

This study provides novel insights into the prognostic significance of further in-depth histological classification in penile malignancies, with a focus not only on HPV positivity but also on differences between Usual-SCCi and Variant-SCCi, as well as its components. The 2016 and subsequent 2022 WHO pathological classifications of penile carcinomas into HPV-related and non-HPV-related subtypes were attributable to pioneering studies, which demonstrated a high prevalence of the virus in carcinomas with basaloid and warty features. Conversely, HPV was found to be negative or infrequently positive in keratinizing SCC variants [18,19]. The notable finding of the current study is the significantly better prognosis observed in patients with Variant-SCCi, as is demonstrated by the longest CSS, against all the other histological categories. It is important to note that the prognosis within the Variant-SCCi group varied considerably depending on its constituent histological components. This heterogeneity has been partially suggested in prior literature [20]. Nevertheless, this report is the first to compare each of the HPV-independent SCC types against each other and against SCCa. Historically, limited attention has been paid to the overwhelming predominance of Usual-SCCi within the HPV-independent group. However, an accurate and up-to-date understanding of the prognostic landscape—including rarer histological subtypes, such as papillary or sarcomatoid carcinoma, even if sparsely represented in large databases, such as SEER—may contribute meaningfully to the optimization of clinical management. While there is consensus that HPV is a major risk factor for penile cancer, there are still ongoing arguments on the prognostic significance [21,22]. There are data supporting better prognoses in penile SCC patients with the presence of high-risk HPV infection [23,24,25]. Some studies have also suggested that SCCa may have a better prognosis compared to HPV-independent SCCs [26]. This seems to be convincing, as this is in line with reports on other malignancies associated with HPV, such as head, neck, and anal, and with the meta-analysis, which confirmed these findings [21,27,28]. However, conflicting results have also been reported, and there is more focus on the differences in responses to treatment or histologic subtypes, even within each HPV-independent SCC and SCCa cancers [26]. The present findings further question the prognostic relevance of the HPV status itself.

Another intriguing finding of the present study is the marked temporal shift in the prognosis of different SCC histologies across the study period. Notably, the 5-year cancer-specific survival (CSS) of SCCa was the poorest among the five histological subtypes in the earlier half of the study period yet improved to the second best in the later half. Several potential explanations may account for this observation. First, there was a notable imbalance in sample sizes between the earlier (*n* = 31) and later (*n* = 179) halves of the cohort. Although not statistically significant, the earlier group included a higher proportion of patients with advanced-stage disease. In addition, patients in the earlier half were significantly more likely to have received chemotherapy (29.0% vs. 12.8%, *p* = 0.041) or radiotherapy (25.8% vs. 5.6%, *p* = 0.001), potentially indicating more aggressive disease or a greater proportion of patients deemed unfit for surgery. These differences highlight the need for caution when interpreting these results. We recognize that diagnostic coding changes could potentially influence the outcomes of SCCa. However, the ICD codes specific to penile cancer, including its variant subtypes, have remained consistent, even after the transition from ICD-O-2 to ICD-O-3 in January 2018. In our study, the SCCa cohort was identified based on histological subtypes; therefore, we believe that coding revisions are unlikely to have affected the observed survival trends. When tumor stage and treatment distributions were further analyzed by era, a gradual reduction in distant and unknown-stage presentations was observed over time, accompanied by an increase in localized and regional disease. It is also important to note that HPV-based histological classification was only formally introduced and widely adopted after 2016. Prior to this, SCCa and SCCi were often not distinctly recognized in either clinical practice or pathology reporting, leading to potential inconsistencies in earlier data. The 2016 WHO classification established clearer diagnostic boundaries, which not only improved reporting accuracy but also likely increased clinical recognition and screening of HPV-associated penile cancer. This heightened awareness and standardization may have contributed to the increased detection and apparent improvement in outcomes observed in the post-2016 era. Given that molecular-targeted therapies and other novel treatment approaches have been developed for HPV-related malignancies in other organs, their potential application to SCCa also warrants further exploration [29,30]. Second, the poor prognoses observed in SCCa and sarcomatoid SCCs in the earlier half of the cohort became less pronounced in the later half. In contrast, verrucous carcinoma consistently demonstrated favorable prognosis across both time periods. This observation likely reflects the intrinsically indolent nature of verrucous tumors rather than improvements in treatment modalities [20]. While the absolute number of verrucous cases remained limited, it was nonetheless greater than that of rarer subtypes, such as papillary or sarcomatoid SCC. Given the rarity of these histological subtypes and the limited statistical power for subtype-specific analyses, fine-grained classification may have limited practicality in both clinical and research settings. It may be reasonable to consider isolating verrucous SCC as a distinct prognostic category while grouping the remaining subtypes—not limited to papillary or sarcomatoid but including Usual-SCCi and SCCa—as a single analytical cohort, especially for survival analyses. Although conducting adequately powered studies for each rare histological variant is likely unfeasible, identifying clinically meaningful differences across broader histologic groupings could provide valuable insights for treatment stratification.

There are limitations to be noted in this study. First, utilizing the SEER database is retrospective in nature. The lack of detailed information, including systemic treatments, comorbidities, chemotherapy regimens, surgical margin status, and p16 immunohistochemistry (IHC), which serves as a surrogate marker for HPV positivity and plays an important role in diagnostic classification or pathological review, presents significant constraints on our analysis. To assess the potential influence of misclassification, we conducted a sensitivity analysis assuming that 10% of the Usual-SCCi cases were actually HPV positive. The results remained consistent, indicating that our findings are robust despite possible coding inconsistencies and the absence of molecular confirmation. Additionally, data regarding the extent and indication of lymph node dissection were unavailable for the majority of the cases, which may have influenced the assessment of oncological outcomes [31]. We utilized CSS as our outcome of interest, as certain patient comorbidity data were lacking, posing questions about the reliability of the overall survival interpretation [32]. The proportion of SCCa in the present cohort appears to be lower than that previously reported in the literature [1]. Although no definitive explanation can be provided, one possible reason is the nature of the SEER database, which collects data from selected geographic regions within the United States and may not fully represent the national or global population distribution [33]. The relatively small sample sizes of the sarcomatoid and papillary subgroups may have reduced the statistical power to detect differences in oncological outcomes. Therefore, these results should be interpreted with caution. Additionally, the continuously evolving classification of histological types can impact the consistency and interpretation of our data. Despite these challenges, analyzing such a large database offers valuable insights for conditions, like penile cancer, where conducting prospective trials is challenging.

## 5. Conclusions

Clear prognostic differences were observed among HPV-independent subtypes, with Variant-SCCi showing significantly more favorable outcomes than Usual-SCCi. Within HPV-associated subtypes, further heterogeneity was evident, particularly highlighting the superior prognosis of verrucous carcinoma. Over time, the prognostic implications of these histological categories appeared to shift, with improved outcomes observed for SCCa in the later study period.

## Figures and Tables

**Table 1:** Patient characteristics.

		**All**	**Usual-SCCi**	**Variant-SCCi**	**SCCa**	***p***
*n*		6332	5706 (90.1)	416 (6.6)	210 (3.3)	
**Year of Diagnosis (%)**	2000–2005	1432 (22.6)	1325 (23.2)	96 (23.1)	11 (5.2)	<0.001
	2006–2010	1320 (20.8)	1198 (21.0)	102 (24.5)	20 (9.5)	
	2011–2015	1569 (24.8)	1430 (25.1)	95 (22.8)	44 (21.0)	
	2016–2021	2011 (31.8)	1753 (30.7)	123 (29.6)	135 (64.3)	
**Age (Median [IQR])**		68.00 [57.00, 77.00]	68.00 [57.00, 77.00]	65.00 [54.00, 75.25]	69.50 [60.00, 78.00]	0.002
**Race (%)**	White	5355 (84.6)	4838 (84.8)	338 (81.2)	179 (85.2)	0.621
	Black	579 (9.1)	516 (9.0)	43 (10.3)	20 (9.5)	
	Asian or Pacific Islander	279 (4.4)	248 (4.3)	24 (5.8)	7 (3.3)	
	American Indian/Alaska Native	59 (0.9)	53 (0.9)	5 (1.2)	1 (0.5)	
	Unknown	60 (0.9)	51 (0.9)	6 (1.4)	3 (1.4)	
**Histology in Detail (%)**	SCC, usual type	5706 (90.1)	5706 (100.0)	0 (0.0)	0 (0.0)	<0.001
	Papillary carcinoma	36 (0.6)	0 (0.0)	36 (8.7)	0 (0.0)	
	Verrucous carcinoma	349 (5.5)	0 (0.0)	349 (83.9)	0 (0.0)	
	Sarcomatoid carcinoma	31 (0.5)	0 (0.0)	31 (7.5)	0 (0.0)	
	Warty carcinoma	14 (0.2)	0 (0.0)	0 (0.0)	14 (6.7)	
	Basaloid carcinoma	195 (3.1)	0 (0.0)	0 (0.0)	195 (92.9)	
	Clear cell carcinoma	1 (0.0)	0 (0.0)	0 (0.0)	1 (0.5)	
**EOD (%)**	Localized	3560 (56.2)	3148 (55.2)	314 (75.5)	98 (46.7)	<0.001
	Regional	2273 (35.9)	2092 (36.7)	82 (19.7)	99 (47.1)	
	Distant	238 (3.8)	221 (3.9)	6 (1.4)	11 (5.2)	
	Unknown	261 (4.1)	245 (4.3)	14 (3.4)	2 (1.0)	
**Surgery (%)**	No/Unknown	634 (10.0)	601 (10.5)	19 (4.6)	14 (6.7)	<0.001
	Local	1815 (28.7)	1598 (28.0)	154 (37.0)	63 (30.0)	
	Partial	2963 (46.8)	2650 (46.4)	201 (48.3)	112 (53.3)	
	Total	686 (10.8)	647 (11.3)	24 (5.8)	15 (7.1)	
	Extensive	234 (3.7)	210 (3.7)	18 (4.3)	6 (2.9)	
**Radiation (%)**	No/Unknown	5784 (91.3)	5198 (91.1)	394 (94.7)	192 (91.4)	0.041
	Yes	548 (8.7)	508 (8.9)	22 (5.3)	18 (8.6)	
**Chemotherapy (%)**	No/Unknown	5612 (88.6)	5041 (88.3)	393 (94.5)	178 (84.8)	<0.001
	Yes	720 (11.4)	665 (11.7)	23 (5.5)	32 (15.2)	
**Follow-up (Months, Median [IQR])**		39.00 [13.00, 95.00]	39.00 [13.00, 95.00]	63.50 [25.00, 129.25]	25.00 [11.25, 47.00]	<0.001

Results are expressed as medians ([interquartile ranges]) or numbers (%). EOD, extent of disease; HPV, human papillomavirus; SCCa, human-papillomavirus-associated squamous cell carcinoma; Variant-SCCi, HPV-independent squamous cell carcinoma variant subtypes; IQR, interquartile range; SCC, squamous cell carcinoma; Usual-SCCi, usual-type squamous cell carcinoma.

**Table 2:** Univariable analysis of factors associated with CSS.

Variable	HR	CI (95%)	*p*
**Age (Continuous)**	1.01	1.01–1.02	<0.001
**Year of Diagnosis**			
2001–2005	(Ref.)		
2006–2010	1.07	0.92–1.24	0.364
2011–2015	1.13	0.98–1.31	0.103
2016–2021	1.21	1.04–1.41	0.011
**Race**			
White	(Ref.)		
Black	1.2	1.02–1.42	0.028
Asian	0.9	0.69–1.16	0.420
Native American/Alaskan Native	1.13	0.69–1.85	0.623
Unknown	0.14	0.03–0.56	0.005
**Histology**			
Usual-SCCi	(Ref.)		
SCCa	0.88	0.63–1.21	0.460
Verrucous	0.22	0.14–0.32	<0.001
Papillary	0.65	0.27–1.30	0.245
Sarcomatoid	1.81	0.96–3.09	0.067
**EOD Total**			
Local	(Ref.)		
Regional	3.16	2.82–3.55	<0.001
Distant	14.2	11.90–16.95	<0.001
Unknown	2.07	1.59–2.70	<0.001
**Surgery**			
Local	(Ref.)		
Partial	1.79	1.55–2.07	<0.001
Total	3.33	2.79–3.98	<0.001
Extensive	3.54	2.77–4.51	<0.001
None/Unknown	4.12	3.44–4.92	<0.001
**Radiation**			
No/Unknown	(Ref.)		
Yes	2.49	2.17–2.86	<0.001
**Chemotherapy**			
No/Unknown	(Ref.)		
Yes	3.14	2.78–3.54	<0.001

In analyses involving subgroups with limited sample sizes (*n* ≤ 50), Firth’s penalized likelihood correction was utilized to mitigate potential small-sample bias and improve the model’s stability. CI, confidence interval; CSS, cancer-specific survival; EOD, extent of disease; HPV, human papillomavirus; SCCa, human-papillomavirus-associated squamous cell carcinoma; Variant-SCCi, HPV-independent squamous cell carcinoma variant subtypes; IQR, interquartile range; SCC, squamous cell carcinoma; Usual-SCCi, usual-type squamous cell carcinoma.

**Table 3:** Multivariable analysis of factors associated with CSS.

Variable	HR	CI (95%)	*p*
**Age (Continuous)**	1.02	1.01–1.02	<0.001
**Year of Diagnosis**			
2001–2005	(Ref.)		
2006–2010	1.14	0.98–1.32	0.085
2011–2015	1.06	0.91–1.23	0.447
2016–2021	1.08	0.93–1.26	0.334
**Race**			
White	(Ref.)		
Black	1.17	0.98–1.38	0.076
Asian	0.86	0.66–1.10	0.230
Native American/Alaskan Native	1.18	0.70–1.85	0.518
Unknown	0.18	0.04–0.52	<0.001
**Histology**			
Usual-SCC	(Ref.)		
HPVa	0.78	0.55–1.06	0.112
Verrucous	0.31	0.20–0.45	<0.001
Papillary	0.82	0.34–1.62	0.594
Sarcomatoid	1.64	0.86–2.80	0.123
**EOD Total**			
Local	(Ref.)		
Regional	2.51	2.21–2.85	<0.001
Distant	8.11	6.61–9.91	<0.001
Unknown	1.53	1.15–2.00	0.004
**Surgery**			
Local	(Ref.)		
Partial	1.16	1.00–1.36	0.055
Total	1.77	1.46–2.13	<0.001
Extensive	1.85	1.43–2.38	<0.001
None/Unknown	2.25	1.86–2.74	<0.001
**Radiation**			
No/Unknown	(Ref.)		
Yes	1.26	1.08–1.46	0.004
**Chemotherapy**			
No/Unknown	(Ref.)		
Yes	1.50	1.29–1.73	<0.001

Analyses were performed using Firth’s penalized likelihood correction to mitigate potential small-sample bias and improve the model’s stability. CI, confidence interval; CSS, cancer-specific survival; EOD, extent of disease; HPV, human papillomavirus; SCCa, human-papillomavirus-associated squamous cell carcinoma; Variant-SCCi, HPV-independent squamous cell carcinoma variant subtypes; IQR, interquartile range; SCC, squamous cell carcinoma; Usual-SCCi, usual-type squamous cell carcinoma.

## Data Availability

The datasets analyzed in this study are publicly available from the Surveillance, Epidemiology, and End Results (SEER) Program of the U.S. National Cancer Institute. Data can be accessed upon reasonable request through the SEER database (https://seer.cancer.gov/), using SEER*Stat software.

## Supplementary Materials

The following supporting information can be downloaded at https://www.mdpi.com/article/10.3390/cancers17223715/s1, Table S1: Additional stratification of the tumor stage and treatment distributions by era. HPV, Human Papillomavirus; SCCa, human papillomavirus-associated squamous cell carcinoma; Variant-SCCi, HPV-independent squamous cell carcinoma variant subtypes; Usual-SCCi, Usual-type squamous cell carcinoma. Figure S1: Cancer-specific survival of the entire population. CI, Confidence Interval; CSS, Cancer-Specific Survival; NR, Not Reached. Figure S2: Sensitivity analysis on cancer-specific survival by five histological groups (Usual-SCCi, SCCa, Verrucous carcinoma, Papillary carcinoma, and Sarcomatoid carcinoma), assuming that random 10% of Usual-SCCi cases were actually HPV-positive. The late decline observed in the Papillary group likely reflects statistical instability due to the limited number of patients at risk in the later follow-up period. Statistical significance is indicated as *** *p* < 0.001. CI, Confidence Interval; CSS, Cancer-Specific Survival; SCCa, human papillomavirus-associated squamous cell carcinoma; Variant-SCCi, HPV-independent squamous cell carcinoma variant subtypes; NR, Not Reached; Usual-SCCi, Usual-type squamous cell carcinoma.
