# Supplementary material for: Histology-Specific Survival in Penile Squamous Cell Carcinoma: A SEER-Based Study Highlighting Human Papillomavirus Status and Prognostic Subtypes
Source: Cancers (Basel). 2025 Nov 20;17(22):3715. doi: 10.3390/cancers17223715 (PMC12651401; doi:10.3390/cancers17223715)
Supplement: Supplementary file 1 [file cancers-17-03715-s001.zip › TableS1.pdf]

The International Classification of Diseases in Oncology, Third Edition (ICD-O-3) codes used for categorization in this study

1. HPV-independent, usual type SCC (Usual-SCCi)

8070, 8071, 8075, 8076

2. HPV-independent, other variant subtypes (Variant-SCCi)

Verrucous carcinoma

8051

Papillary

8050, 8052

Sarcomatoid

8074

3. HPV-associated SCC (SCCa)

Basaloid

8083, 8123

Warty

8054

Clear cell

8084
