# Supplementary figures and images for: Histology-Specific Survival in Penile Squamous Cell Carcinoma: A SEER-Based Study Highlighting Human Papillomavirus Status and Prognostic Subtypes
Source: Cancers (Basel). 2025 Nov 20;17(22):3715. doi: 10.3390/cancers17223715 (PMC12651401; doi:10.3390/cancers17223715)

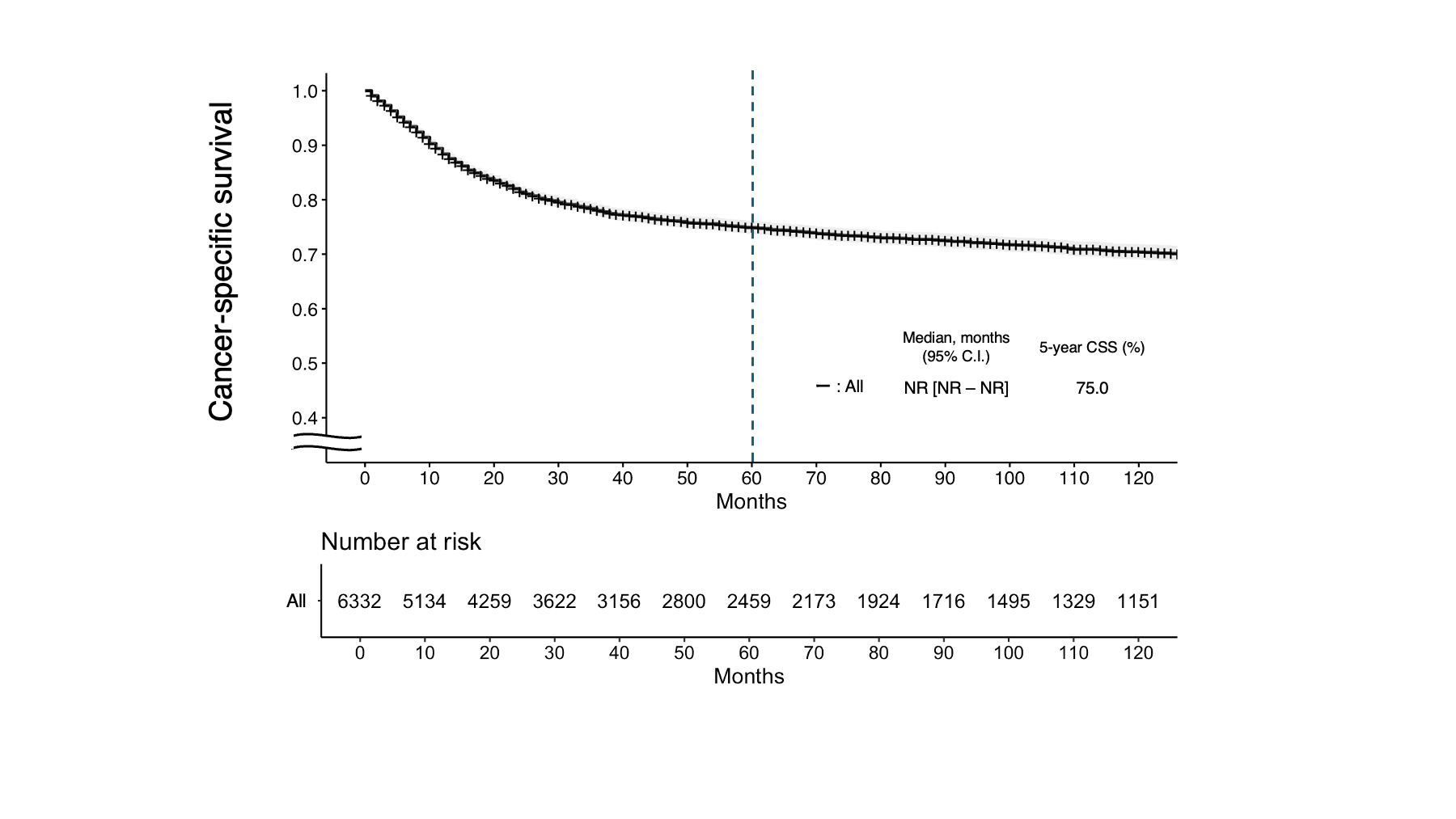

Supplement: Supplementary file 1 [file cancers-17-03715-s001.zip › FigS1.png]

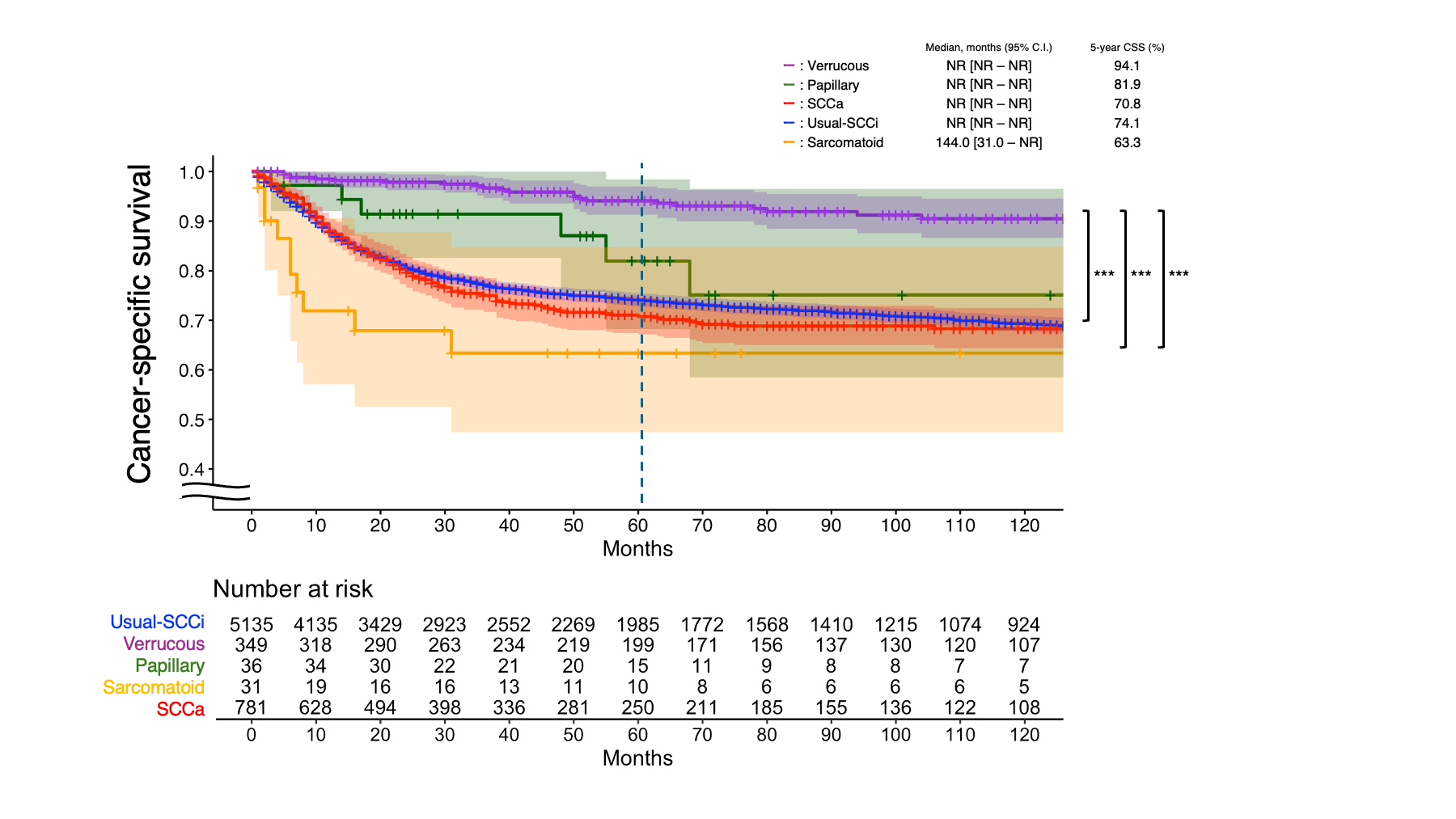

Supplement: Supplementary file 1 [file cancers-17-03715-s001.zip › FigS2.png]
